# Improving Reading in Adolescents and Adults With High-Functioning Autism Through an Assistive Technology Tool: A Cross-Over Multinational Study

**DOI:** 10.3389/fpsyt.2019.00546

**Published:** 2019-08-07

**Authors:** Arlinda Cerga-Pashoja, Jorge Gaete, Antoneta Shishkova, Vesna Jordanova

**Affiliations:** ^1^Faculty of Population Health, London School of Hygiene and Tropical Medicine, London, United Kingdom; ^2^Central and North West London NHS Foundation Trust, London, United Kingdom; ^3^Department of Public Health and Epidemiology, Faculty of Medicine, Universidad de los Andes, Santiago, Chile; ^4^Millennium Nucleus to Improve the Mental Health of Adolescents and Youths (Imhay), Santiago, Chile; ^5^Parallel World Association, Plovdiv, Bulgaria; ^6^Institute of Psychiatry, Neurology and Neurosciences, King’s College London, London, United Kingdom; ^7^Neurodevelopmental Services, South London and Maudsley NHS Foundation Trust, London, United Kingdom

**Keywords:** autism spectrum disorder, adults, adolescents, Natural Language Processing, reading

## Abstract

People with autism spectrum disorder (ASD) experience reading comprehension difficulties, often misinterpreting complex texts, metaphors, and idioms. We have developed and tested a new assistive technology tool for adaptive, personalized text simplification, called Open Book. This tool is an open-sourced, online platform that uses Natural Language Processing with the specific aim of assisting reading and aiding understanding of written text for people with ASD. The accessibility and effectiveness of Open Book was tested by examining the differences in text comprehension scores between the original texts and texts that were simplified by Open Book tool, randomly allocated to study participants. Two hundred forty-three participants (153 adults and 90 adolescents) with high-functioning ASD were recruited in the UK, Spain, and Bulgaria. Regarding the primary outcome, results showed that both adults and adolescents with ASD gave more correct answers for the simplified (*M* = 11.2, SD = 4.1) than original texts (*M* = 10, SD = 4.1; *p* < 0.001). This finding was consistent across age groups and countries. Regarding the secondary outcome, when participants were asked to blindly rate how easy was to understand each text, simplified texts were rated as easier (*M* = 7.6, SD = 2.4) to understand than the original texts (*M* = 8.7, SD = 2.6; *p* < 0.001). The Open Book software seems to have the potential to be a useful tool in assisting reading among people with ASD. Our findings support our primary hypothesis that texts simplified through Open Book were easier to comprehend compared to original texts.

## Introduction

The autism spectrum disorder (ASD) has been recognized as the fastest growing developmental disability with 1 in 88 children diagnosed having ASD (1). People with ASD experience a range of language deficits, which have a life-long impact on their psychosocial functioning (2). These deficits include difficulties in comprehension of speech and writing, especially misinterpreting and understanding complex instructions (3). Although individuals at the higher end of the autistic spectrum appear to have good reading abilities, several studies have shown that these individuals have difficulties in different components of written language comprehension. For instance, they fail to make inferences about social scripts and understand metaphors, which interfere with successful social communication (4). Many individuals with ASD are unable to derive the gist or meaning of written documents (5–7). Studies show that people with high-functioning ASD have excellent phonetic decoding (ability to capture the meaning of unfamiliar words by translating groups of letters back into the sounds that they represent, link them to one’s verbal vocabulary, and access their meaning) but poor comprehension (6, 8, 9). Similar results were reported by Huemer and Mann (10) who compared reading accuracy with reading comprehension in a population with ASD. This study found that error patterns observed in the participants suggested that children with ASD are more focused on accurately decoding text than on preserving the meaning of the passage. This was supported in another study where readers with ASD were good at decoding sounds but had poor comprehension (11). These findings also support the evidence that the skill in both decoding and linguistic comprehension is necessary if skill in reading is to advance (12). In addition, people with ASD are not able to use their background knowledge to construct an understanding of text (13).

Traditionally, the difficulty with reading comprehension has been related to the cognitive profile of these readers especially with their problems to comprehend the perspectives of others (14). Saldana and Frith (15) have found that people with ASD have difficulty with inferences, which appear to be greater in text with social content and suggest that these difficulties may be related to mentalizing deficits and could also influence other reading processes such as referential inferences or attributions of authors’ aims. Furthermore, comprehension difficulties have been associated with differences in linguistic information processing causing a negative impact in the metaphor comprehension (16).

Several problems with the pragmatic aspects of language have been found among people with ASD (16, 17). For instance, Dennis et al. (4) studied the different ability to understand pragmatic inferences about given or presupposed knowledge in mental state words. This study confirmed that children with high-functioning ASD struggle to understand metaphors and make inferences about social scripts. These results are also consistent with those of Beversdorf et al. (18) who showed that people with high-functioning ASD recall less of emotional sentences than nonemotional ones. On the other hand, recent evidence suggest that the risk for reading comprehension difficulties is a specific characteristic of the social-communication phenotype of many high functioning ASD children and adolescents (19–22).

Although there is an abundance of research on reading difficulties for children with autism, there seems to be a considerable gap in investigation of this issue beyond adolescence. Nevertheless, a few studies that address language disorders in adults with autism indicate that, although reading accuracy improves with age in high functioning children with autism, they continue to struggle with many linguistic phenomena such as homographs, multiple meaning words, phrases, and metaphors (10).

To the knowledge of the authors, there are no reading comprehension interventions tested among adults with autism, and there are very few studies involving adolescents. In a recent review about reading comprehension interventions for school-aged children and adolescents with ASD (23), 12 studies were identified, 3 using treatment comparison designs and 8 using single-case designs. These interventions included strategy instruction (24–27), explicit instruction (28–30), and anaphoric cueing (6, 31). None of these interventions have been tested using an experimental design or including a large sample. However, these interventions were time consuming and required a facilitator, which increased the cost of the intervention (23). The field of reading interventions for people with ASD had followed the research involving students with reading difficulties in general (32, 33), and most of the interventions tested for students with ASD have included reading expert recommendations (34). However, it seems that there is high need for research-based knowledge to enhance reading comprehension performance in people with ASD, especially among older adolescents and adults (23).

Assistive technology has been used to enhance communication and academic skills for children with disabilities (35, 36). The use of technology to teach several academic and social skills to students with ASD has a long history, since the first study reporting the use of a computer to increase understanding of how letters and sounds form words, and how texts can form expressions (37, 38). However, very few studies have explored or tested the use of assistive technology to facilitate reading comprehension among ASD subjects (39).

The assistive tool tested in this study was developed in the project FIRST (Flexible Interactive Reading Support Tool) by a multinational group of interdisciplinary researchers that involved collaboration between clinical, machine-learning, and Natural Language Processing (NLP) experts in the UK, Spain, and Bulgaria. We adapted Language Technologies resources to design a system called Open Book in three languages—English, Spanish, and Bulgarian. Further details of this project can be found in previous publications (40–42).

Open Book is a noncommercial electronic platform that can be personalized to meet and support the specific reading needs of people with autism. It uses Natural Language Processing (NLP) to make documents for people with autism more accessible. Some of the processes utilized by Open Book include the following: detection of language obstacles in the text; adding definition to terms or infrequent (rare) words; adding images to words in order to aid word visualization; providing synonyms for infrequent words; providing options to change text format (e.g., background color, text color); and “magnify” feature which highlights particular sentence to ease focusing users’ attention and support when following specific text sections. This approach is supported by several studies saying that text comprehension depends on understanding words and integrating their meaning into a mental model of the text (43–45).

Open Book can convert a standard document into a personalized and simplified version, which was hypothesized that it would be easier to understand. Another feature of the platform is that it encompasses two different interfaces—for independent users with autism and for caregivers such as parents or teachers. The Open Book independent user can benefit from assistive elements using features such as “Explain word,” “Explain with image,” “Provide summary,” or “Ask caregiver” to make the text clearer. The program also simplifies complex text structures by shortening long sentences and clarifies ambiguities. Not relying purely on textual changes, the conversion software also provides illustrative pictures to selective words and offers concise document summaries.

The interface designed for caregivers provides them with a semiautomatic program where they cannot only convert text using the NLP technologies implemented in the software but can also make their own editions to the text. They can upload images, review texts from their user’s library, suggest other support if needed, and/or create new documents. All the documents are collected in the user’s personal library, which can be arranged with different folders and labels. A privacy function allows the user to keep select documents private and not share them with their caregiver.

The initial software prototype was produced in English, Spanish, and Bulgarian.

The aim of this study was to assess the accessibility, utility, and the effectiveness of Open Book in simplifying complex texts by making them easier to understand for adolescents and adults with high-functioning ASD in UK, Bulgaria, and Spain.

The hypothesis was that texts simplified through Open Book would be easier to comprehend compared to original texts for participants with ASD. It was expected that, when participants were tested about written texts’ comprehension, they would give more correct responses on the simplified texts compared to original (not-simplified) documents. It was also hypothesized that participants would blindly rate simplified texts as easier to comprehend compared to original texts.

By improving access of people with autism to written information, we ultimately aim to facilitate their empowerment and social inclusion. Open Book is expected to help individuals with autism to increase their independence by improving access to the wealth of textual information that is available in the information society.

## Material and Methods

### Study Design

Crossover design was used to test (46–48) the effectiveness of Open Book to improve reading comprehension among adolescents and adults with autism spectrum disorder.

### Participants

All participants who met the following criteria were included in the study: a) a formal ICD-10 diagnosis of ASD based on diagnostic clinical interview conducted by psychiatrists or clinical psychologists; b) 12–17 years old in the adolescents branch of the study undertake in Spain and Bulgaria, and ≥18 years old in the adult branch of the study carried out in the UK and Spain; and c) a score of ≥70 in a measure of an intelligence test confirmed by clinical records. The study exclusion criteria were as follows: a) not native speakers of the respective languages, i.e., English, Spanish, and Bulgarian; b) documented history of learning disabilities; c) additional diagnosis of dementia or other organic brain disorder that could affect memory; and d) presence of a sensory impairment that could prevent reading, writing, or hearing.

### Ethical Approval

All study procedures were in accordance with the ethical standards of the respective institutional and/or national research committees and with the 1964 Helsinki declaration and its later amendments or comparable ethical standards.

Full ethical approval for the project was sought and received from each center separately.

In the UK, full ethical approval was sought and received by East of Scotland Research Ethics Service (ref: 13/ES/0059). Separate ethical approvals were also received by local Research and Development teams from each NHS site that participated in recruitment.

In Bulgaria, Parallel World received approval from the Ethical Commission of Plovdiv University St. Paisii Hilendarski. In addition, for the control group, permissions were received from the school management where the tests were conducted. Parallel World is a Registered Administrator of Personal Data according to the Bulgarian Law for Protection of the Personal Data.

In Spain, consultations were conducted following internationally accepted ethical regulations, the legal normative applicable, and the Good Clinical Practice standards (CPMP/ICH/135/95). The guidelines of investigation compatible with those suggested by the American Psychological Association for investigations involving human participants were also followed.

The process for obtaining participant informed consent was in accordance with the REC guidance and GCP. All participants provided written informed consent. The decision regarding participation in the project was entirely voluntary. The research worker emphasized to participants that consent regarding project participation could be withdrawn at any time without penalty or affecting the quality or quantity of their future medical care, or loss of benefits to which the participant was otherwise entitled. No project-specific testing was done before informed consent had been obtained.

The informed consent forms were signed and dated by all potential participants/parents before they entered the project. The research worker explained the details of the project and provided a participant information sheet, then allowed participants to consider whether they liked to be involved in the project. The research worker encouraged the participant to ask any questions that could help them make a decision on their potential involvement in the project.

Informed consent was collected from each participant before they underwent the reading comprehension test, including history taking related to the project. One copy of the informed consent form was kept by the participant, while the other was kept by the research worker and was retained in the project Master File.

The study was granted by the FP7 EU Grant for Social Inclusion.

### Sample Size

The sample size calculation was based on the precision with which we will be able to estimate the proportion of participants who prefer the simplified text. Based on a clinical assumption that 80% of people with ASD would prefer the simplified text, and using a confidence level of 95%, a sample of 100 participants would allow us to have 80% power to estimate the true proportion that prefer the simplified text of between 72 and 88%.

### Recruitment

Recruitment involved active collaboration between the clinicians in the specialist clinical centers and service user and carers.

The recruitment in the UK was expanded at a national scale including several important urban areas such as Greater London, Leicester, Sheffield, and Plymouth. Majority of the participants were recruited from the National Health Service (NHS). Voluntary and charity organizations also played a very important role in reaching recruitment targets. Thus, the National Autistic Society played a major role in recruitment activity in the UK.

In Spain, the recruitment was focused in the whole province of Madrid, and it involved specialized diagnostic and treatment centers, public and private schools, centers for work mediation for people with ASD, and leisure facilities for people with ASD.

Although the autism diagnostic assessment provision in Bulgaria is sporadic, we have developed a successful collaborative work with clinical centers who have autism expertise in Sofia and Stara Zagora and Parallel World Association (charity organization) in Plovdiv.

All participant services across the three countries used identical recruitment strategy.

A researcher arranged to see the adults and the parents of children with ASD who expressed an interest in participating in the project. Consent was given by adult participants, and for children, it was obtained by their parents.

A total of 243 people who met the inclusion criteria completed the study. A detailed description of the participants is provided in **Table 2**.

### Randomization

Reading comprehension testing that was conducted in a controlled environment under exam conditions.

One hundred fifty-three participants set reading tests in groups of 20 participants. Each participant received three simplified and three original documents. Participants were blind to text conditions. Both participants and researchers were blinded to text allocation sequence, which was block randomized by an independent researcher in the UK using a 1:1 ratio.

### Materials

The reading comprehension tests for adults used documents that covered a range of topics: education about general and mental health, sexual health issues, newspapers articles, chapters from electronic novels, and general knowledge articles. The texts for adolescents were selected through children and young books, school material, and the Internet.

**Text selection**. Each clinical center in UK and Bulgaria identified 12 texts that were appropriate to reading abilities and interests of respective age groups (adolescents and adults). The research team in Spain identified 24 texts in total: 12 for adolescents and 12 for adults. Texts for adults were selected from comprehension test batteries used to examine reading comprehension in language proficiency, e.g., International English Language Testing System (IELTS) and Cambridge English Proficiency. All texts identified by clinical teams were inspected and analyzed by Natural Language Processing (NLP) specialists, partners in the FIRST project. NLP specialists selected 6 out of 12 texts in each language, which were matched between languages for word length, complexity, and number of obstacles. Thus, each adult text used in Spain was matched for word length and complexity with the texts used in the UK. The same was done for adolescent texts in Spain and Bulgaria.

**Text simplifications**. The original texts were forwarded to the technical teams who uploaded them into Open Book and simplified them automatically. The outcome was postedited by the clinical teams through Open Book caregiver platform. Reading obstacles and their resolutions are described in **Table 1**.

**Table 1:** Reading obstacles and resolutions.

Obstacle	Resolution
Multiple copulative coordinated clauses	Substitute with sentences divided by periods.
Long sentences	Sentences < 15 words
Semicolon and suspension points	Avoid the use of semicolon and suspension points
Brackets and uncommon punctuation marks (&,%,/…)	Avoid uncommon punctuation marks
Improper grammar	Correct grammar
Polysemy	Avoid using easier synonym. Detect and highlight when domain is not clear
Phraseological units (idioms, Lexicalized metaphors)	Substitute by a simple word. Highlight when substitution is not possible
	Provide simple definitions to explain phraseological units
Slang	Substitute infrequent slang with simpler synonym
	Provide simple definitions to explain slang
Infrequent acronyms and abbreviations	Expand infrequent acronyms and abbreviations
Temporal adjectives	Disambiguate temporal adjectives
Anaphors	Resolve all types of anaphors when possible. Leave anaphors with low resolution confidence level.
Non-lexicalized metaphors	Provide idea of inferred meaning when possible and highlight
Long paragraphs	Divide long paragraphs
Complex/infrequent words	Substitute infrequent words with simpler synonym
	Provide simple definitions to explain infrequent words

### Measures

#### Primary Outcome: Comprehension Score

**Figure 1:**
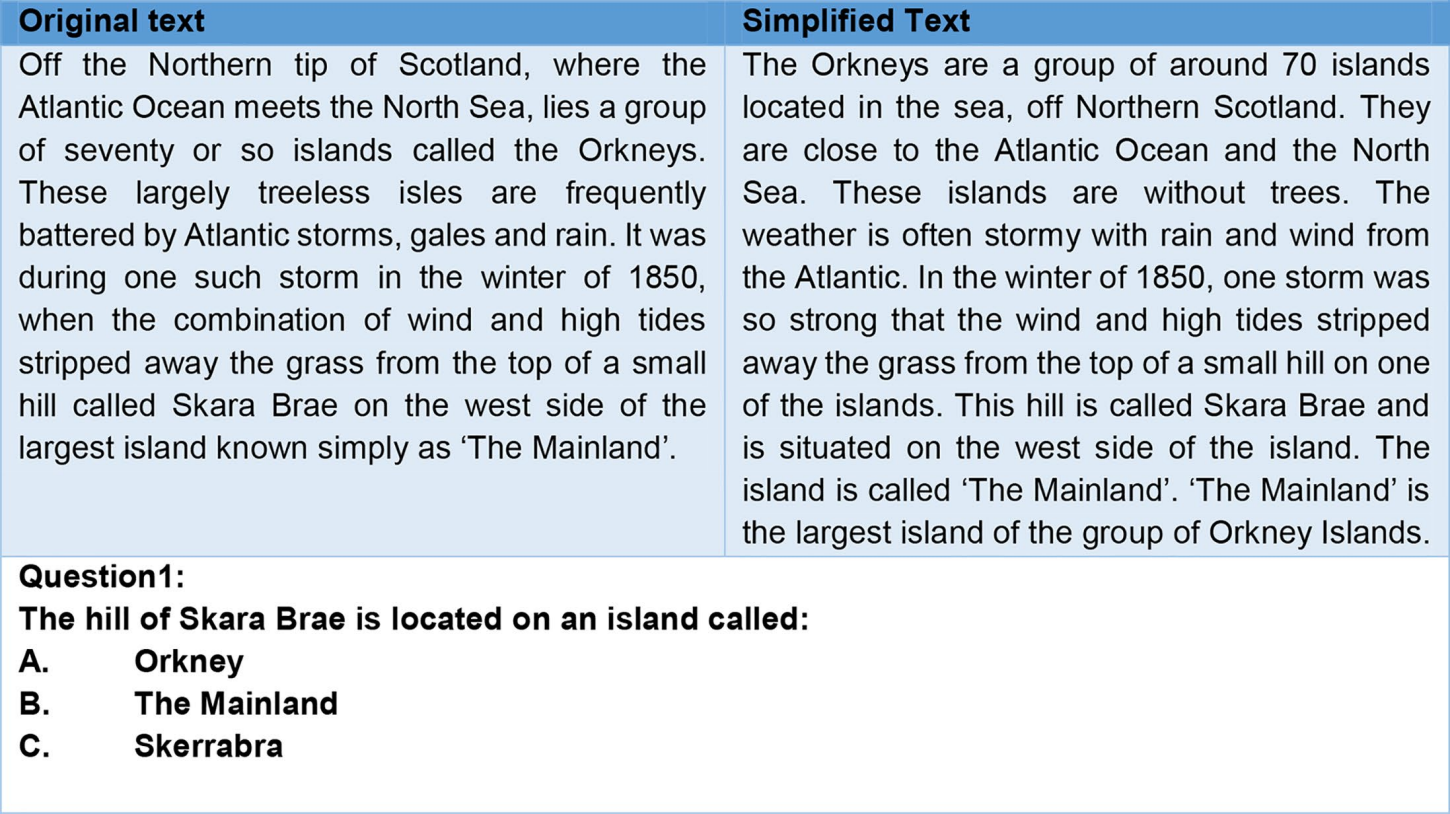
Example of two text versions followed by a multiple-choice question (MCQ).

The study participants undertook a reading comprehension test under exam conditions. Multiple choice questions (MCQs) were generated by each clinical team for their respective texts, with the help of technical partners’ input. MCQs were selected based on the original texts so that they could tap into the general comprehension of the text’s content, especially parts of the text with identified obstacles. The MCQs were the same for both original and simplified texts, and an example of two text versions followed by the MCQ is provided in **Figure 1**.

Each adult text was followed by six MCQs, and each adolescents’ texts had four MCQs. This selection was done to accommodate adolescents’ performance within the same timeframe as the adults.

Each center created a library of 12 texts, 6 original and 6 modified (simplified) version of original texts, while the MCQs were the same for each corresponding text. The test battery was comprised of three original and three simplified texts randomly selected for each participant. Both adolescents and adult participants were given 10 min to read each text and answer all MCQs per text.

The primary outcome was the comprehension score calculated by adding the text scores for each question. Scores from the simplified texts were compared with scores from the original texts. Adult texts were followed by six questions each. Every right answer was scored as 1, and each wrong answer was scored as 0. Therefore, each text score could range from 0 (no correct answer) to 6 (all correct answers) for adults, and 0–4 for adolescents. The overall score for original and simplified texts was calculated separately by adding the score for each of the three corresponding texts. The overall range of scoring values are 0–18 for adults (6 questions × 3 texts) and 0–12 for adolescents (4 questions × 3 texts).

##### Secondary Outcome: Self-Reported Text Complexity

The secondary outcome was self-reported text complexity that was measured on a Likert-type scale, where participants were asked to blindly rate how easy it was to understand each text. The scores ranged from 1 (very easy) to 5 (very difficult). Therefore, the range of scores for each text was 1–5, and overall (for three texts) 3–15. Higher subjective scores indicated self-reported higher level of comprehension difficulty, while lower scores indicated that the texts were easier to understand.

### Data Analysis

**General features**. Descriptive statistics are presented as numbers and percentages for categorical variables and means with standard deviations for continuous data.

**Primary analyses for primary and secondary outcomes**. The primary analyses tested the effectiveness of the tool using repeated measures *t*-tests for primary and secondary outcomes. The effect size using the Cohen’s *d* was also calculated (49).

**Secondary analyses**. Correlation analyses were performed to assess the association between original and simplified text scores and subjective rating to test if participants were able to identify which text was original and which one was simplified. The scores of the MCQ tests were compared between the original and simplified versions of each text and between the individuals. Paired *t*-tests for analyses of comparisons between the original and simplified texts and independent sample *t*-tests for comparisons between individuals were used. Finally, univariable and adjusted regression analyses were performed to assess the association between participants´ characteristics and simplified text scores.

All data were stored electronically and analyzed with SPSS.

## Results

### General Features

**Figure 2:**
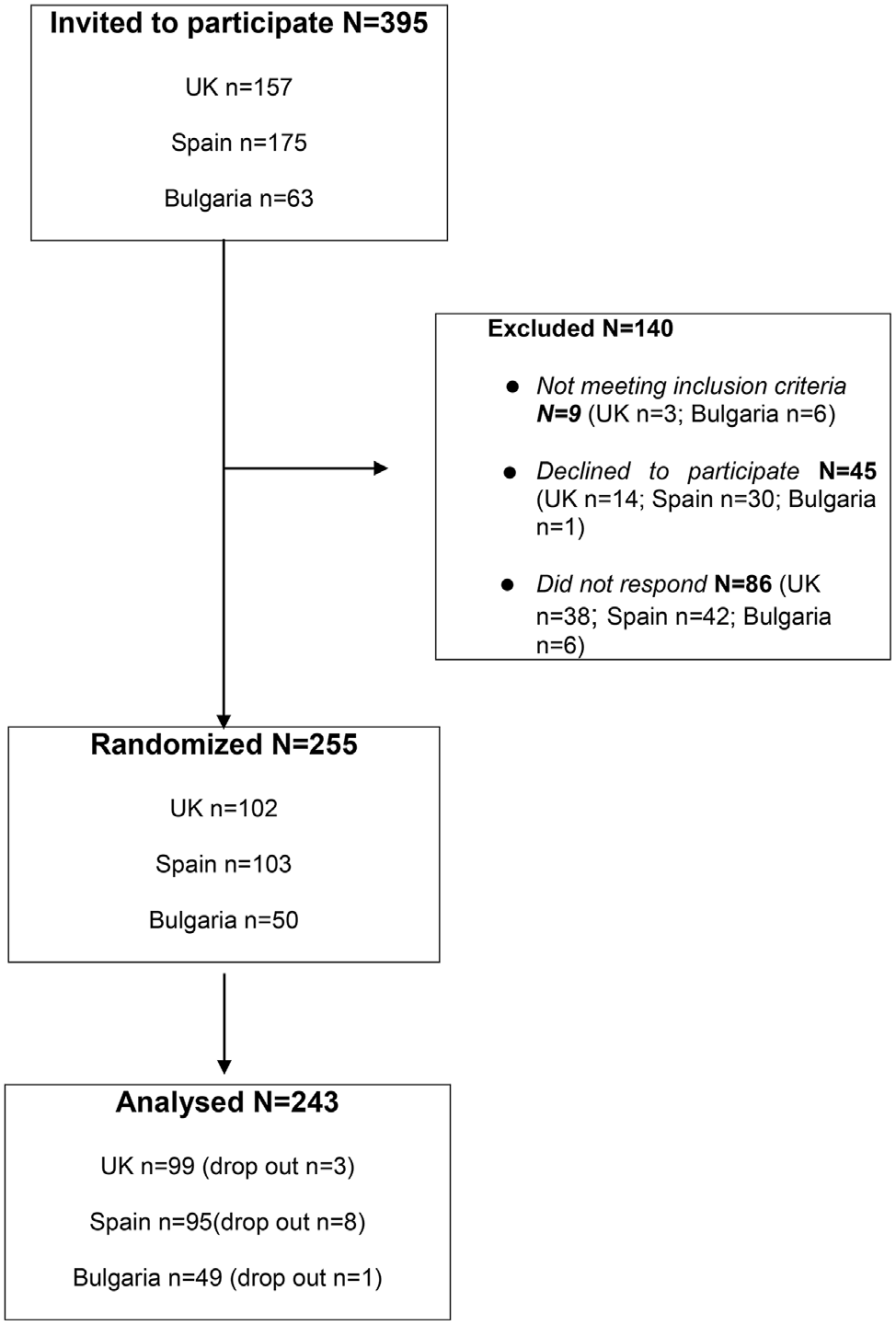
Participant flow diagram.

We invited 445 people to participate in the evaluation task, 140 of whom were excluded because they did not meet inclusion criteria, declined to participate, or did not respond to our invitation. Three hundred five people consented to participate; 11 of them dropped out and did not carry out the reading test. The main reason for the drop out was poor health on the day of the test. Two hundred ninety-four people completed the test, and all their data were analyzed. For detailed information, see **Figure 2**.

A total of 243 subjects (29%, female) participated in this study. Overall age ranged from 12 to 70 years old [adolescents, mean = 14.0 years old (SD = 2.1); adults, mean = 35.3 years old (SD = 13.1)]. The sample was predominantly male. Considering the moderately homogenic ethnic composition of Bulgaria and Spain, the sample was principally (93%) of white ethnic background. Adult participants had higher IQ scores [109.25 ± 21.4 (75–168)] than adolescent participants [85.97 ± 13.2 (70–127)] *p* < 0.001.

A prominent characteristic of our adult participants sample is that they were well educated with just one person educated to elementary level (see **Table 2**). More than half of the sample were educated to secondary school level (55.7%) followed by graduates (35.57%), and MSc and PhD holders (4.03%, respectively). Nevertheless, although adult participants are very well educated, high percentages are unemployed, single, and do not live independently (see **Table 2**).

**Table 2:** Participants’ characteristics.

Participant group	Adults (*n* = 153)	Adolescents (*n* = 90)
	Mean (SD) or frequency (%)	Mean (SD) or frequency (%)
Age	35.3 (13.1)	14.0 (2.1)
Gender		11.2 (4.1)
Male	114 (74.5)	
Female	39 (25.5)	
Ethnicity		
White	140 (91.5)	85 (93.4)
Black	4 (2.7)	–
Asian	3 (2)	2 (2.2)
Mix	3 (3)	–
Other	2 (1.7)	–
IQ score	109.25 ± 21.4 (75–168)	85.97 ± 13.2 (70–127)
ADHD Diagnosis	17 (11.1%)	13 (14.3%)
Special Education Needs	8 (5.2%)	34 (37.4%)
Education		
Mainstream-School	22 (14.4%)	45 (49.5%)
Mainstream-School with Support	41 (26.8%)	23 (25.3%)
Home tuition	–	6 (6.6%)
Highest education level achieved (only adults)		
Elementary	1 (0.7)	
Secondary	83 (55.7)	
University	53 (35.6)	
PhD	6 (4.0)	
MSc	6 (4.0)	
Occupation (only adults)		
Student	41 (27.0)	
Professional	12 (7.9)	
Manager	2 (1, 3)	
Clerical and Intermediate	10 (6.6)	
Technical and craft	9 (5.9)	
Manual labor	16 (10.5)	
Unemployed	58 (38.2)	
Retired	4 (2.6)	

Psychiatric comorbidities were prevalent in our adult sample, especially depression (25.5%) and anxiety (23.5%), but no psychiatric comorbidities were identified among adolescents.

### Primary Analyses Results

#### Primary Outcome: Comprehension Score

The scores in **Table 3** indicate the summary of the results of correct answers to the MCQs for the original and simplified texts. The scores ranged from 0 to 18 for adults’ texts and 0–12 for adolescents’ texts, with each score meaning a correct answer to a question related to text comprehension. The two sets of text scores were compared through related *t*-tests. All participants had a higher score on the simplified texts than on the original texts, meaning that overall both adults and adolescents gave more correct responses for simplified texts compared to original texts. This difference was statistically significant in all groups, with the exception among adolescents in Bulgaria. When all participants were included in the analysis, difference in the scores for simplified texts (*M* = 11.2, SD = 4.1) and original texts (*M* = 10, SD = 4.1) conditions was statistically significant (*p* < 0.001, effect size = 0.3). Among different groups, the effect sizes were of medium magnitude (*d* = 0.3–0.7). These findings were also consistent across age groups. Examining age groups separately, adults performed better on questions about simplified texts (*M* = 13.3, SD = 3.3) compared to original texts (*M* = 12, SD = 3.5; *p* < 0.001, *N* = 153). Adolescents also gave more correct responses on questions about simplified content (*M* = 7.8, SD = 2.8) compared to questions about original texts (*M* = 6.6, SD = 2.6; *p* < 0.001, *N* = 90).

**Table 3:** Text score analysis.

Participant group	N	Original Mean (SD)	Simplified Mean (SD)	Difference in means (95% CI)	*p* value	Effect size *d*
Adults and adolescents Overall	243	10.0 (4.1)	11.2 (4.1)	1.2 (0.9, 1.6)	<0.001	0.3
Adults	153	12.0 (3.5)	13.3 (3.3)	1.3 (0.8, 1.8)	<0.001	0.4
Adolescents	90	6.6 (2.6)	7.8 (2.8)	1.1 (0.7, 1.6)	<0.001	0.4
UK adults	99	12.3 (3.9)	13.8 (3.7)	1.5 (0.8, 2.2)	<0.001	0.4
Spain adults and adolescents	95	9.3 (3.5)	10.6 (3.2)	1.3 (0.8, 1.7)	<0.001	0.4
Spain adults	54	11.5 (2.6)	12.4 (2.1)	1.0 (0.3, 1.7)	0.009	0.4
Spain adolescents	41	6.5 (2.1)	8.1 (2.8)	1.7 (1.2, 2.2)	<0.001	0.7
Bulgaria adolescents	49	6.8 (2.9)	7.4 (2.9)	0.7 (−0.1, 1.4)	0.08	

#### Secondary Outcome: Self-Reported Text Complexity

A similar set of analyses were performed for the participants’ blind rating about text complexity. Overall, all participants blindly rated simplified texts as easier to understand than the original texts. This difference was statistically significant in all groups, with the exception among adolescents in Spain. When all participants were included in the analysis, the original text was considered more difficult to understand (*M* = 7.6, SD = 2.4) than the simplified text (*M* = 8.7, SD = 2.6; *p* < 0.001, *N* = 243). The findings were consistent for our subgroups of adults and adolescents. See **Table 4**.

**Table 4:** Analysis of subjective scoring.

Participant group	N	Original Mean (SD)	Simplified Mean (SD)	Difference (*) Mean (95% CI)	*p*-value	Cohen’s *d*
Adults and adolescents	243	8.7 (2.6)	7.6 (2.4)	−1.0 (−1.3, −0.7)	<0.001	0.4
Adults	153	9.1 (2.3)	8.0 (2.2)	−1.2 (−1.6, −0.8)	<0.001	0.5
Adolescents	90	7.8 (2.9)	7.0 (2.7)	−0.8 (−1.3, −0.3)	0.001	0.3
UK adults	99	9.3 (2.3)	8.0 (2.1)	−1.3 (−1.8, −0.8)	<0.001	0.6
Spain adults and adolescents	95	8.1 (2.4)	7.3 (2.4)	−0.8 (−1.2, −0.3)	0.001	0.3
Spain adults	54	8.7 (2.4)	7.8 (2.3)	−0.9 (−1.5, −0.3)	0.006	0.4
Spain adolescents	41	7.3 (2.3)	6.7 (2.4)	−0.7 (−1.4, 0.1)	0.07	0.3
Bulgaria adolescents	49	8.3 (3.2)	7.3 (3.0)	−0.9 (−1.6, −0.3)	0.008	0.3

### Secondary Analyses Results

#### Association Between Text and Self-Reported Text Complexity Scores

The correlation coefficients and *p* values between the original text and subjective scores was 0.03 (*p* = 0.56) and between the simplified text and subjective scores was 0.03 (*p* = 0.67).

#### Association Between Participants’ Characteristics and Simplified Text Scores

The univariable and adjusted regression analyses between participants’ characteristics and simplified text scores are presented in **Table 5**. The majority of variables examined were associated with the simplified text scores in the univariable analyses. The exception was occupation and ADHD, which were not found to be significant. Female participants scored higher than male participants, with scores 1.6 units higher. Participants with higher IQ values achieved higher text scores on simplified texts. A 10-unit increase in IQ was associated with a 0.9-unit increase in text score. A higher level of education was also associated with higher outcome values. Those with university education had scores that were 6.6 units higher, on average, than those with no or only elementary education. There was little difference in scores between married and divorced/widowed participants. However, single participants had the highest scores.

**Table 5:** Univariable and multivariable regression models.

Variable	Category	N	Mean (SD)	Univariable models		Adjusted model	
				Coefficient (95% CI)	*p* value	Coefficient 95% CI)	*p* value
Gender	Male	193	10.9 (4.1)	0	0.02		
	Female	50	12.5 (4.2)	1.6 (0.3, 2.8)			
ADHD	No	204	11.4 (4.1)	0	0.26		
	Yes	29	10.5 (3.7)	−0.9 (−2.5, 0.7)			
Psychiatric diagnosis	No	180	10.5 (3.8)	0	<0.001		
	Yes	49	14.1 (3.6)	3.6 (2.4, 4.8)			
IQ (*)	–	–	–	0.9 (0.7, 1.2)	<0.001		
Education	None/elementary	25	7.4 (10.6)	0	<0.001	0	0.04
	Secondary	144	10.6 (3.7)	3.2 (1.7, 4.7)		0.8 (−0.8, 2.3)	
	University	65	14.0 (3.2)	6.6 (5.0, 8.2)		2.1 (0.2, 4.1)	
Marital status (†)	Married	59	11.1 (4.1)	0	0.007		
	Divorced/widow	16	11.4 (4.5)	0.3 (−1.8, 2.4)			
	Single	115	12.9 (3.4)	1.8 (0.6, 3.0)			
Occupation (†)	Unemployed/retired	68	12.6 (3.7)	0	0.51		
	Student	41	12.5 (2.9)	−0.1 (−1.6, 1.4)			
	Employed	76	11.9 (4.4)	−0.7 (−2.0, 0.6)			

(*) Regression coefficient given for a 10-unit increase in IQ; (†) Data not applicable for Spanish adolescents.

In the multivariable analyses, the results suggested that higher education was significantly associated with the text scores.

## Discussion

The study provides the first clinical evaluation of novel assistive technology, Open Book, that aims to assist reading comprehension of written texts in adults and adolescents with ASD. While this is not a reading comprehension intervention *per se*, we have found that Open Book can help convert written texts into simpler forms, which are easier to understand by people with ASD. Open Book can be used either autonomously or with the online aid of a carer or teacher, which makes the tool adaptable to different ages and levels of comprehension. Open Book is available in English, Spanish, and Bulgarian. It automatically simplifies written text by splitting long sentences; replacing metaphors, slangs, and idioms with commonly used synonyms; resolving anaphors, etc. It also has the option of replacing some complex words with pictures, which was especially used by adolescents and their teachers.

Open Book was evaluated by adults and adolescents in UK, Spain, and Bulgaria. Significant work went towards developing reading comprehension testing methodology and materials that were age specific and matched for the level of complexity across three languages.

The evaluation of Open Book indicates that adult and young people with ASD benefit from automatic text simplification. Participants in our study achieved significantly better tests’ results when they processed simplified than original texts, which indicates that their understanding of the text content was enhanced when the written information was modified by the assistive technology.

The effect sizes were of medium magnitude overall, and for the adolescent sample in Spain, the effect size was large. The subjective, blind ratings of self-reported text complexity indicated in all instances that simplified versions were deemed as easier to comprehend compared to original texts.

Advanced education (university studies vs. lower education) was associated with higher text scores. We may hypothesize that reading skills improve with education, but it may be explained by having better cognitive abilities. However, other findings support the idea that individuals with ASD continue to struggle with complex linguistic phenomena (10).

There are some limitations in this study. Even though Open Books seems to have a positive impact in immediate reading comprehension of written texts, we are not able to determine if there is a longer-term effect in the reading abilities of our target group. Furthermore, we could not evaluate the effect of the use of this assistive technology in the functionality of our participants and their quality of life. Although we have demonstrated the potential benefits for high-functioning individuals, the results may not be generalizable to other people on the autistic spectrum.

## Conclusions

The study indicates that assistive technologies could be useful in supporting understanding of written text for people with ASD. The written texts simplified by the Open Book platform were significantly easier to understand by both adults and adolescents with high functioning ASD. This demonstrates a novel direction in translational autism research that opens the doors of interdisciplinary collaboration and innovation to benefit people with this disabling condition.

The next step would be to assess the feasibility of Open Book, its uptake and utility by both people with ASD and their carers in real-life conditions.

## Ethics Statement

All study procedures were in accordance with the ethical standards of the respective institutional and/or national research committees and with the 1964 Helsinki declaration and its later amendments or comparable ethical standards. Full ethical approval for the project was sought and received from each centre separately. In the UK full ethical approval was sought and received by East of Scotland Research Ethics Service (ref: 13/ES/0059). Separate ethical approvals were also received by local Research and Development teams from each NHS site that participated in recruitment. In Bulgaria Parallel World received approval from the Ethical Commission of Plovdiv University St. Paisii Hilendarski. Also, for the control group, permissions were received from the School management where the tests were conducted. Parallel World is a Registered Administrator of Personal Data according to the Bulgarian Law for Protection of the Personal Data. In Spain consultations were conducted following internationally accepted ethical regulations, the legal normative applicable and the Good Clinical Practice standards (CPMP/ICH/ 135/95). The guidelines of investigation compatible with those suggested by the American Psychological Association for investigations involving human participants were also followed.

## Author Contributions

AC-P, AS and VJ designed and executed the study, assisted with the data analyses and wrote de paper. JG assisted with the data analyses and collaborated with the writing of the results and the whole paper.

## Funding

The study was granted by the FP7 EU Grant for Social Inclusion. This study was supported by the Central and North West London NHS Foundation Trust, with assistance from M. Keats and A. Bela. The writing contribution of the author JG was supported by Millennium Science Initiative of the Ministry of Economy, Development and Tourism, grant “Millennium Nucleus to Improve the Mental Health of Adolescents and Youths, Imhay.”

## Conflict of Interest Statement

The authors declare that the research was conducted in the absence of any commercial or financial relationships that could be construed as a potential conflict of interest.
